# Onset of Alzheimer disease in apolipoprotein ɛ4 carriers is earlier in butyrylcholinesterase K variant carriers

**DOI:** 10.1186/s12883-024-03611-5

**Published:** 2024-04-09

**Authors:** Roger M. Lane, Taher Darreh-Shori, Candice Junge, Dan Li, Qingqing Yang, Amanda L. Edwards, Danielle L. Graham, Katrina Moore, Catherine J. Mummery

**Affiliations:** 1 Ionis Pharmaceuticals, 2855 Gazelle Court, Carlsbad, CA 92010 USA; 2 Department of Neurobiology, Care Sciences and Society, Center for Alzheimer Research, Division of Clinical Geriatric, Karolinska Institutet, Stockholm, Sweden; 3 Biogen, Cambridge, MA USA; 4 Dementia Research Centre, Institute of Neurology, London, UK

**Keywords:** Butyrylcholinesterase, Cholinergic, Glial activation, Innate immune, Apolipoprotein E, Amyloid, Tau, Sex differences

## Abstract

**Background:**

The authors sought to examine the impact of the K-variant of *butyrylcholinesterase* (*BCHE-K*) carrier status on age-at-diagnosis of Alzheimer disease (AD) in *APOE4* carriers.

**Methods:**

Patients aged 50–74 years with cerebrospinal fluid (CSF) biomarker-confirmed AD, were recruited to clinical trial (NCT03186989 since June 14, 2017). Baseline demographics, disease characteristics, and biomarkers were evaluated in 45 patients according to *BCHE-K* and *APOE4* allelic status in this post-hoc study.

**Results:**

In *APOE4* carriers (*N* = 33), the mean age-at-diagnosis of AD in *BCHE-K* carriers (*n* = 11) was 6.4 years earlier than in *BCHE-K* noncarriers (*n* = 22, *P* < *.*001, ANOVA). In *APOE4* noncarriers (*N* = 12) there was no observed influence of *BCHE-K*. *APOE4* carriers with *BCHE-K* also exhibited slightly higher amyloid and tau accumulations compared to *BCHE-K* noncarriers. A predominantly amyloid, limited tau, and limbic-amnestic phenotype was exemplified by *APOE4* homozygotes with *BCHE-K*. In the overall population, multiple regression analyses demonstrated an association of amyloid accumulation with *APOE4* carrier status (*P* < *.*029), larger total brain ventricle volume (*P* < *.*021), less synaptic injury (Ng, *P* < *.*001), and less tau pathophysiology (p-tau_181_, *P* < *.*005). In contrast, tau pathophysiology was associated with more neuroaxonal damage (NfL, *P* = .002), more synaptic injury (Ng, *P* < *.*001), and higher levels of glial activation (YKL-40, *P* = .01).

**Conclusion:**

These findings have implications for the genetic architecture of prognosis in early AD, not the genetics of susceptibility to AD. In patients with early AD aged less than 75 years, the mean age-at-diagnosis of AD in *APOE4* carriers was reduced by over 6 years in *BCHE-K* carriers versus noncarriers. The functional status of glia may explain many of the effects of *APOE4* and *BCHE-K* on the early AD phenotype.

**Trial registration:**

NCT03186989 since June 14, 2017

**Supplementary Information:**

The online version contains supplementary material available at 10.1186/s12883-024-03611-5.

## Background

The cholinergic hypothesis of AD states that selective loss of cholinergic neurons, arising from basal forebrain nuclei, and decreased levels of the neurotransmitter, acetylcholine (ACh), trigger neurodegeneration and cognitive impairment [1]. Corticolimbic cholinergic denervation may be evident at early stages of AD [2, 3]. The failure of this circuitry is inextricably linked with cognitive deficits in memory, learning, attention, and processing speed [4]. Synaptic release of ACh initiates cholinergic neurotransmission and is rapidly terminated by acetylcholinesterase (AChE). The availability of ACh in cholinergic synapses is deficient in AD and can be increased with acetylcholinesterase inhibitors (AChE-Is) [5]. The cholinergic system produces both rapid focal synaptic signaling and slow diffuse extracellular signaling through alpha 7 nicotinic ACh receptors (α7-nAChRs) that act to control glial cell reactivity and functional state [6]. Glial cells provide homeostasis and neuroprotection of the central nervous system (CNS), and if this functionality is deficient, amyloid-β (Aβ) pathology can accumulate [7]. However, tau tangle pathology is more strongly correlated with glial activation than Aβ pathology, and microglial and astrocyte activation may better predict the spatiotemporal spread of tau tangles [8, 9].

The gene* apolipoprotein E* (*APOE*) encodes for the protein ApoE, which is the major intercellular lipid carrier in the CNS and is primarily produced by astrocytes, reactive microglia, vascular mural cells, and choroid plexus cells [10, 11]. The *APOE4* polymorphism is the major genetic risk factor for sporadic AD [12]. In *APOE4* carriers, functional glial responses to clear Aβ are deficient and favor the accumulation of amyloid pathology [7]. Cerebral amyloid accumulation begins earlier in life in *APOE4* carriers than in noncarriers [13, 14].

Butyrylcholinesterase (BuChE), along with AChE, is involved in the enzymatic breakdown of both synaptic and extracellular Ach [15, 16]. Astrocytes secrete BuChE and the ACh synthesizing enzyme, choline acetyltransferase (ChAT), to maintain a steady state equilibrium of hydrolysis and synthesis of extracellular ACh [17]. In addition to particular populations of neurons in the amygdala and hippocampus [18], BuChE is localized in glia, myelin, and endothelial cells, and continues to increase in concentration with age, especially in the deep cortex and white matter [19]. Aβ, ApoE, and BuChE are prominent constituents of amyloid plaques and interact with each other to influence the catalytic activity of BuChE [20, 21]. For example, CSF ApoE protein profoundly alters the catalytic functioning and stability of CSF BuChE in patients with mild AD in an ApoE concentration- and polymorphism-dependent manner; this interaction is also Aβ concentration-dependent [20, 22]. *BCHE* genotype and CSF BuChE activity are also correlated with markers of glial activation in early AD [23, 24]. Lower BuChE activity is associated with higher amyloid accumulation in patients with mild AD [24].

The most common single-nucleotide polymorphism (SNP) of *BCHE*, the Kalow-variant (*BCHE-K;* 3q26.1-3q26.2; nucleotide G1615A, codon A539T; rs1803274), is carried by 18–35% of individuals in Western populations [25–28]. In *APOE4* carriers, reduced BuChE activity is more marked in *BCHE-K* carriers, with a *BCHE-K* allele dose-dependent reduction in BuChE activity and lowering of glial activation markers [24, 29]. The *BCHE-K* and *APOE4* alleles interact to significantly reduce the age-at-onset of AD [30], and to increase the likelihood of progression from mild cognitive impairment (MCI) to AD [27, 31] and from cognitively unimpaired older individuals to early AD [26]. Carriers of both *APOE4* and *BCHE-K* alleles in the MCI stage of AD have a limbic-amnestic phenotype and progress most rapidly in the mild stage of AD, where they are the only genotype group with a significant response to AChE-I treatment [27, 31–33].

The primary objective of this cross-sectional analysis of early AD patients aged less than 75 years was to evaluate *BCHE-K* effects on age-at-onset of AD in *APOE4* carriers. In addition, this study sought to characterize the phenotypic expression of early AD in carriers of *APOE4* and *BCHE-K* relative to other genotype groups with respect to accumulations of amyloid and tau pathology, neurodegeneration, glial activation, hippocampal atrophy, ventricular expansion, and cognitive function.

## Methods

This trial (NCT03186989) was conducted in accordance with Good Clinical Practice Guidelines of the International Council for Harmonisation and according to the ethical principles outlined in the Declaration of Helsinki, and reporting adhered to Consolidated Standards of Reporting Trials (CONSORT) guidelines as reported previously [34]. CONSORT guidelines are not applicable in this cross-sectional, baseline analysis. The completed study [34] was approved by relevant ethics committees. Written informed consent was provided by all participants.

### Study eligibility criteria

Eligible study participants were between the ages of 50 and 74 years of age; had probable early AD (amnestic or non-amnestic), defined by a Mini-Mental State Examination score (MMSE) of 20–27, inclusive [35], and either a Clinical Dementia Rating Overall Global Score of 1, or a Global Score of 0.5 with a Memory Score of 1 [36]; a CSF pattern of low Aβ_1-42_ (≤ 1200 pg/ml), elevated total-tau (> 200 pg/ml) and p-tau (> 18 pg/ml), and a total-tau to Aβ_1-42_ ratio > 0.28 [37]; and a diagnosis of probable AD based on National Institute of Aging-Alzheimer Association (NIA-AA) criteria [38]. Exclusion criteria included any condition preventing participation in writing tasks, MRI or lumbar puncture (LP); significant risk of suicide, major depression, psychosis, confusional state or violent behavior; clinically significant laboratory, vital sign or electrocardiogram finding; and medical history of brain or spinal disease that would be expected to interfere with CSF circulation.

### Assessments

CSF from patients was analyzed for markers of amyloid accumulation (inversely indexed by Aβ_42_), tau pathophysiology (tau phosphorylated at threonine 181 [p-tau_181_]), neuroaxonal degeneration (neurofilament light chain [NfL]), synaptic injury (neurogranin [Ng]), and glial activation (chitinase-3-like protein 1 [YKL-40]). The CSF analytes and assays are detailed in Table 1. Of 46 patients entering the study, 45 were characterized for common SNPs of *APOE* (ie, *APOE4*, *APOE3*, and *APOE2*)*,* and *BCHE* rs1803274 (ie, *BCHE-K*). Mutations associated with dominantly inherited AD were also assessed (ie, *APP*, *PSEN1*, and *PSEN2*).

**Table 1:** Methods used to assess CSF biomarkers

**CSF analyte**	**Biology indexed**
**Aβ**_**42**_ (β-amyloid 1-42) Elecsys β-amyloid 1-42 CSF performed at Roche Diagnostics, Indianapolis, IN	Parenchymal amyloid accumulation
**p-tau**_**181**_ (tau phosphorylated at threonine 181) Elecsys Phospho-Tau (181P) CSF performed at Roche Diagnostics, Indianapolis, IN	Tau tangle pathology
**NfL**^**a**^ (neurofilament light chain) Uman, performed at Immunologix, Tampa, FL	Neuroaxonal degeneration
**Ng**^**a**^ (neurogranin) Euroimmune, performed at Immunologix, Tampa, FL)	Postsynaptic injury
**YKL-40**^**a**^ (chitinase-3-like protein 1) ELLA (Protein Simple), performed at Immunologix, Tampa, FL)	Microglial and astrocyte activation [39]

^a^All CSF samples for NfL, Ng, and YKL-40 were tested using a single batch of reagents

The study required 3-dimensional (3D) T1-weighted structural magnetic resonance imaging (MRI) scans of the head, and volumetric analyses calculated using VivoQuant™, which is comprised of a preprocessing module and a multi-atlas segmentation module, followed by visual inspection and manual editing, if needed [40]. The mean baseline ventricular volume and hippocampal volume were expressed as a percentage of the total intracranial volume (%TIV). Domains of cognitive functioning were assessed using the MMSE and the Repeatable Battery for the Assessment of Neuropsychological Status (RBANS) [41].

### Statistical analysis

Patient baseline characteristics were summarized according to genotype and sex (Table 2). Quantitative assessments were summarized using descriptive statistics, including number of patients, mean, and standard deviation. Qualitative assessments were summarized using frequency counts and percentages. The exact test was used to examine the Hardy-Weinberg equilibrium (HWE) of the distribution of *APOE* and *BCHE* alleles in the study population. All exact tests were performed using the R package “Hardy Weinberg” [42]. The HWE *P* value measures the strength of evidence against the null hypothesis that the distribution does not follow Hardy-Weinberg equilibrium. A large *P* value is consistent with the distribution following HWE.

**Table 2:** Early AD phenotype across genotype groups defined by *APOE4* and *BCHE-K* carrier status

	**Mean value for variable (SD)**	***2E4 & K* (*N* = 4)**	***E4 & K* (*N* = 11)**	***E4 & No-K* (*N* = 22)**	***No-E4 & K* (*N* = 5)**	***No-E4 & No-K* (*N* = 7)**
**Sex**	Female	75%	64%	50%	40%	29%
**Onset of AD**	Age-at-AD diagnosis (yrs)	60.8^a^ (4.4)	60.6^a1^ (6.1)	67.0^b1^ (4.0)	63.9 (7.6)	62.8 (7.8)
	Age-at-baseline (yrs)	62.0^a^ (4.8)	62.1^a2^ (5.9)	68.3^b2^ (4.0)	64.8 (7.3)	64.4 (6.6)
	Time since AD diagnosis at baseline (yrs)	1.2^a^ (1.2)	1.5^b^ (1.3)	1.3^a^ (1.2)	0.9^a^ (0.7)	1.6^b^ (1.9)
**Neuroimaging**	Hippocampal vol., % of ICV	0.26^a^ (0.05)	0.26^a^ (0.05)	0.24^a^ (0.03)	0.28^b^ (0.02)	0.28^b^ (0.03)
	Ventricular vol., % of ICV	1.8^a^ (0.73)	2.9^a^ (1.54)	2.8^b^ (1.23)	2.3^b^ (0.97)	2.9^a^ (1.15)
**CSF markers**	Aβ_42_, pg/mL	554^a^ (111)	641^a^ (139)	676^a^ (193)	755^b^ (195)	831^b^ (176)
	p-tau_181_, pg/mL	34.0^b^ (11.8)	40.4^a^ (12.2)	38.7^b^ (14.8)	42.5^a^ (15.8)	40.4^a^ (13.5)
	NfL, pg/mL	1003^b^ (265)	1392^a^ (498)	1222^b^ (395)	1316^b^ (160)	1458^a^ (366)
	Ng, pg/mL	493^b^ (188)	568^a^ (188)	506^b^ (257)	540^a^ (245)	515^b^ (247)
	YKL-40, ng/mL	190^b^ (86.0)	247^b^ (143.3)	247^b^ (85.0)	351^a^ (164.5)	293^a^ (168.5)
**Cognition**	MMSE Total (0–30)	24.3^b^ (2.5)	23.8^b^ (2.5)	23.5^a^ (2.3)	23.4^a^ (2.4)	23.9^b^ (2.3)
	MMSE Memory (0–6)	4.0^a^ (1.4)	3.9^a^ (1.0)	4.1^a^ (1.1)	4.2^a^ (1.3)	4.9^b^ (1.2)
	MMSE Visual Construction (0–1)	0.8^b^ (0.5)	0.7^b^ (0.5)	0.7^b^ (0.5)	0.8^b^ (0.4)	0.3^a^ (0.5)
	RBANS Total (40–160)	72.8^b^ (8.3)	65.5^a^ (10.5)	67.5^b^ (12.4)	72.0^b^ (13.4)	66.0^a^ (11.8)
	RBANS Delayed Memory (40–154)	56.0^a^ (19.3)	52.7^a^ (13.7)	47.8^a^ (5.9)	69.0^b^ (26.2)	64.3^b^ (21.5)
	RBANS Visuospatial/Constructional (40–154)	101.8^b^ (28.8)	90.4^b^ (25.6)	85.4^b^ (19.8)	79.4^a^ (21.1)	80.3^a^ (23.9)

*ICV* Intracranial volume, *MMSE* Mini-Mental Status Examination, *RBANS* Repeatable Battery for the Assessment of Neuropsychological Status, *SD* Standard deviation

^1^ = significant difference, *P* < *.*001, ANOVA, *P* = .001, ANCOVA; ^2^ = significant difference,  *P* =.001, ANOVA, *P* =.002, ANCOVA

^a^Numerically more pathology or more clinical impairment

^b^Numerically less pathology or less clinical impairment

An analysis of variance (ANOVA) and an analysis of covariance (ANCOVA) were used to test whether the mean age-at-diagnosis of AD (primary analysis) or the mean age-at-baseline differed across two or more genotype groups, ie, by *BCHE-K* carrier status in *APOE4* carriers, and in *APOE4* homozygotes and heterozygotes. When the ANCOVA model was applied, the model included *BCHE-K* carrier status and sex as factors, and baseline MMSE total score as covariate. Prior to performing ANOVA and ANCOVA, the normality assumption of residuals was tested using the Kolmogorov-Smirnov test. If significant departures from normality were observed, the Wilcoxon Rank Sum test was applied. Both ANOVA and ANCOVA were applied to test baseline CSF Aβ_42_ across two or more genotype groups. When ANCOVA was applied, age-at-baseline was included as an additional covariate. If the normality assumption was not satisfied, both the ANOVA and ANCOVA models were fitted to the log-transformed data. Box plots were used to visualize data by group.

Relationships between CSF Aβ_42_, CSF p-tau_181_, other CSF biomarkers, and brain volumes (as %TIV) were explored in the overall population and in each genotype group in a simple linear correlation analysis with a Pearson correlation coefficient. The squared Pearson correlation coefficient (*R*^2^) and *P* value were provided in the correlation analysis and interpreted by descriptors to indicate the strength of the relationship. Correlation coefficients were defined as: 0.81 ≤ *R*^2^ < 1 as strong; 0.49 ≤ *R*^2^ < 0.81 as moderately strong; 0.25 ≤ *R*^2^ < 0.49 as moderate; 0.09 ≤ *R*^2^ < 0.25 as weak; and *R*^2^ < 0.09 as negligible. Scatterplots with a simple linear regression line were produced to depict the relationships between two quantitative variables.

Multiple regression analysis was also used to assess the functional relationships between the biomarker of interest and amyloid and tau pathophysiology. A multiple regression model was applied with CSF Aβ_42_ and CSF p-tau_181_ as the response variables and *APOE4*, *BCHE-K*, age-at-baseline, sex, baseline MMSE total score, and the biomarker of interest (ie, CSF p-tau_181,_ Aβ_42_, NfL, Ng, YKL-40, total hippocampal volume, and total brain ventricular volume) as independent variables. This determined the strength of association of CSF Aβ_42_ or CSF p-tau with parameters of interest, in conjunction with the other independent variables included in the model. Cognitive domains were assessed on the MMSE and the RBANS [41].

## Results

The study was conducted at 12 centers in Canada, Finland, Germany, the Netherlands, Sweden, and the UK between August 2017 and February 2020. One hundred and two patients were assessed for eligibility. Of these, 56 were excluded based on the eligibility criteria, and one enrolled patient did not provide genetic test results. The study sample comprised 45 patients with a mean age of 65.8 ± 5.8 years and a mean baseline MMSE total score of 23.6 ± 2.3.

The *APOE4* allele was present in 73% (*N* = 33) of the patients: 22% (*N* = 10) homozygotes and 51% (*N* = 23) heterozygotes [43]. The *BCHE-K* allele was present in 36% (*N* = 16) of patients; 7% (*N* = 3) homozygotes and 29% (*N* = 13) heterozygotes (Table S1). Of the 16 *BCHE-K* carriers, 69% (*N* = 11) were *APOE4* carriers (Table 2). All non-*APOE4* alleles were *APOE3*, except for one patient with an *APOE2/3* genotype who also carried one *BCHE-K* allele. The distribution of *APOE* genotypes (HWE exact *P* value = 1) and *BCHE* genotypes (HWE exact *P* value = 0.376) were consistent with Hardy-Weinberg equilibrium. One patient had an autosomal dominant *PSEN2* mutation, and was heterozygous for *APOE4* and *BCHE-K*.

### Age-at-diagnosis of AD in *APOE4* carriers was reduced in carriers of *BCHE-K*

There were no significant *APOE4* carrier or allele frequency-associated differences in age-at-diagnosis of AD or in age-at-baseline (i.e., age at the time of this cross-sectional investigation) [43]. In contrast, *BCHE-K* homozygotes (*n* = 3) had a lower mean age-at-diagnosis of 59.4 years versus heterozygotes (*n* = 13) of 62.2 years, versus noncarriers (*n* = 29) of 66.0 years (Table S1; *P* = 0.048, ANOVA).

**Fig. 1:**
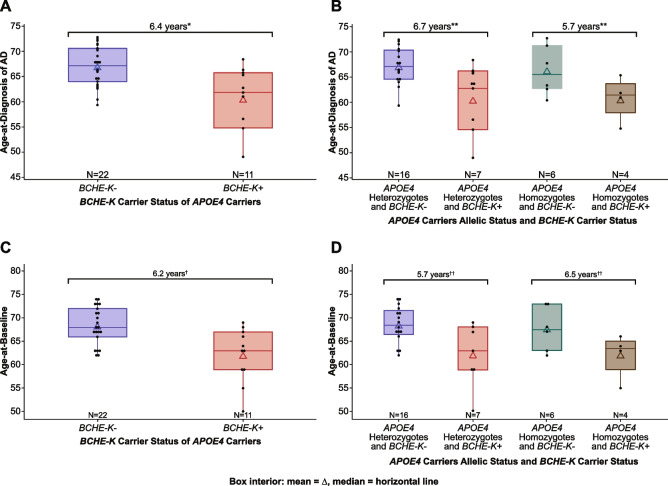
Age-at-diagnosis of AD and age-at-baseline by *BCHE-K* carrier status in *APOE4* carriers, homozygotes, and heterozygotes. **A** Age-at-diagnosis of AD in *APOE4* carriers by *BCHE-K* carrier status. **B** Age-at-diagnosis of AD in *APOE4* homozygotes and heterozygotes by *BCHE-K* carrier status. **C** Age-at-baseline of study in *APOE4* carriers by *BCHE-K* carrier status. **D** Age-at-baseline of study in *APOE4* homozygotes and heterozygotes by *BCHE-K* carrier status. * *P* < *.*001, ANOVA; *P* = .001, ANCOVA with *BCHE-K* carrier status and sex as factors and baseline MMSE total score as covariate for mean age-at-diagnosis of AD. **P = .013, ANOVA; *P* = .019, ANCOVA with *APOE4* homozygotes and heterozygotes by *BCHE-K* carrier status and sex as factors and baseline MMSE total score as covariate for mean age-at-diagnosis of AD. † *P* = .001, ANOVA; *P* = .002, ANCOVA with *BCHE-K* carrier status and sex as factors and baseline MMSE total score as covariate for mean age-at-study-baseline. †† *P* = .015, ANOVA; *P* = .025, ANCOVA with *APOE4* homozygotes and heterozygotes by *BCHE-K* carrier status and sex as factors and baseline MMSE total score as covariate for mean age-at-study-baseline

In the primary analysis of this investigation, *APOE4* carriers with *BCHE-K* alleles (*n* = 11) showed a significantly lower mean age-at-diagnosis (*n* = 22) (60.6 ± 6.1 versus 67.0 ± 4.0; *P* < 0.001, ANOVA; *P* = 0.001, ANCOVA) (Fig. 1A; Table 2), and mean age-at-baseline (62.1 ± 5.9 versus 68.3 ± 4.0; *P* = 0.001, ANOVA; *P* = 0.002, ANCOVA) compared to *APOE4* carriers without *BCHE-K* alleles (Fig. 1C). The mean age-at-diagnosis of AD in *BCHE-K* carriers versus noncarriers was also significantly different from *APOE4* heterozygotes (6.7 years; 60.5 versus 67.3 years) and homozygotes (5.7 years; 60.8 versus 66.4 years) (*P* = 0.013, ANOVA; *P* = 0.019, ANCOVA) (Fig. 1B). Significant differences were also seen in age-at-baseline (Fig. 1D).

In *APOE4* noncarriers (*N* = 12), the mean age-at-diagnosis and age-at-baseline of the study were similar between *BCHE-K* carriers (*n* = 5) at 63.9. ± 7.6 and 64.8 ± 7.3 years, respectively, and noncarriers (*n* = 7) at 62.8 ± 7.8 and 64.4 ± 6.6 years, respectively (Table 2).

### In patients with both *APOE4* and *BCHE-K*, younger mean age-at-baseline was accompanied by increased amyloid and tau accumulations

Across genotype groups defined by *APOE4* and *BCHE-K* carrier status, the proportion of female patients fell as the burden of *APOE4* and *BCHE-K* alleles diminished (Table 2). Carriers of both alleles had the earliest age-at-diagnosis, greater memory deficits, and the most amyloid accumulation. *APOE4* carriers with *BCHE-K* had slightly higher amyloid and tau accumulations than *APOE4* carriers without *BCHE-K*, despite a mean age-at-baseline that was 6.2 years earlier. In *APOE4* carriers, neurodegeneration and glial activation were similar between carriers and noncarriers of *BCHE-K* (Table 2).

### Patients with both *APOE4* and *BCHE-K* had the most amyloid pathology and presented a limbic-amnestic phenotype

Three of four (75%) *APOE4* homozygotes with *BCHE-K* were female. *APOE4* carriers exhibited more of a temporo-limbic (hippocampal atrophy > ventricular expansion) and amnestic (memory > visuospatial impairment) phenotype relative to *APOE4* noncarriers (Table 2). *APOE4* carriers relative to noncarriers had higher levels of amyloid pathology (inversely indexed by the CSF Aβ_42_ = 664 ± 175 versus 799 ± 180 pg/mL, respectively; *P* = 0.028 ANOVA, *P* = 0.028 ANCOVA) (Data not shown [43]). Carriers of both *APOE4* and *BCHE-K* had higher levels of amyloid pathology (CSF Aβ_42_ = 641 ± 139 pg/mL); amyloid pathology was further increased in *APOE4* homozygotes with *BCHE-K* (CSF Aβ_42_ = 554 ± 111 pg/mL). The latter subgroup has only the ApoE4 protein, and despite the highest levels of Aβ accumulation, this subgroup had the lowest levels of tau pathophysiology, synaptic injury, neuroaxonal damage, glial activation, and ventricular expansion (Table 2).

### Patients without *APOE4* and *BCHE-K* had the least amyloid pathology and an amnestic-sparing phenotype

In marked contrast to carriers of both *APOE4* and *BCHE-K*, noncarriers of these alleles displayed less hippocampal atrophy and an amnestic deficit-sparing phenotype, with the lowest levels of amyloid pathology, the highest levels of neuroaxonal injury, and high levels of ventricular expansion (Table 2). Noncarriers relative to carriers of both *APOE4* and *BCHE-K* had higher CSF indices of tau pathophysiology, synaptic injury, glial activation, and multidomain cognitive deficits, but memory impairments were less severe (Table 2).

### More amyloid pathology correlated with less tau pathophysiology, especially in carriers of *APOE4* and *BCHE-K*

**Fig. 2:**
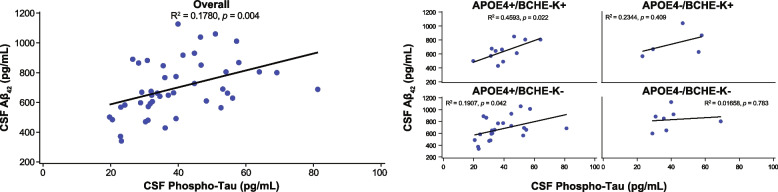
Correlations of CSF Aβ_42_ versus CSF p-tau_181_ in the overall population and in *APOE4* and *BCHE-K* subgroups. Simple linear correlation analyses: *R* square and *P* value were obtained by fitting a simple linear regression model

**Fig. 3:**
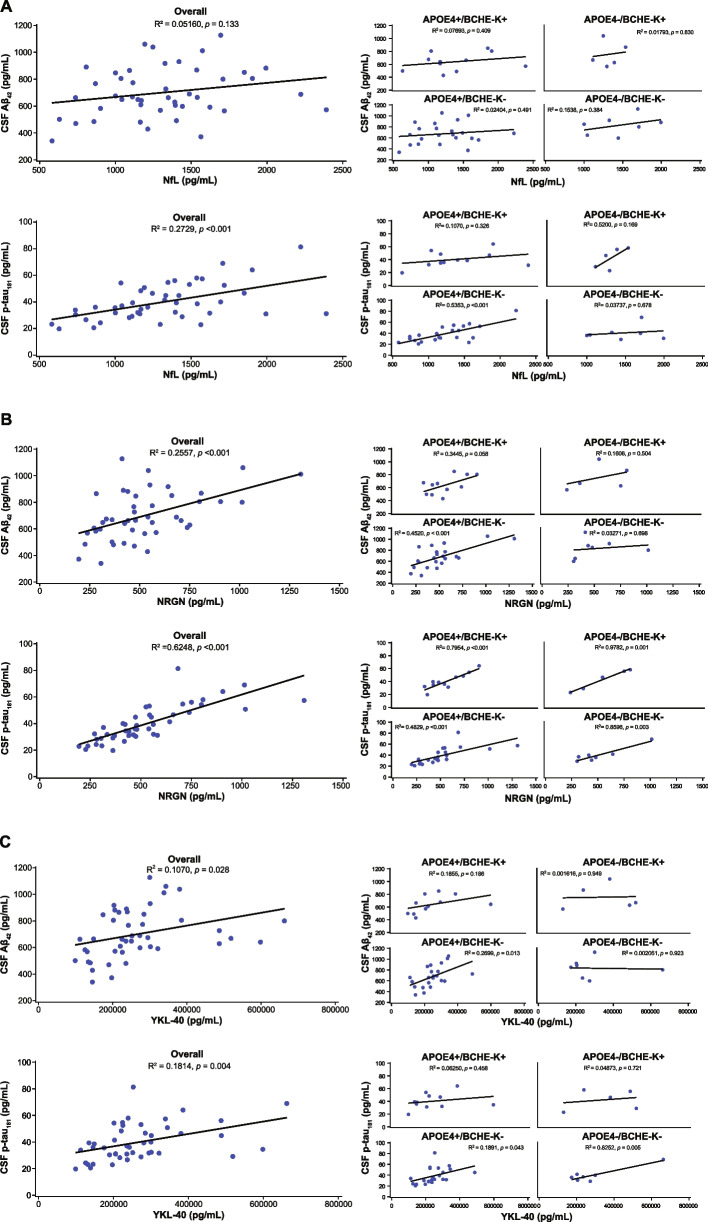
Correlations of neurodegenerative and glial activation markers with CSF Aβ_42_ and CSF p-tau_181_ in the overall study population and in genotype groups. **A** CSF neurofilament light chain (NfL) (higher levels index more neuroaxonal injury). **B** CSF neurogranin (Ng) (higher levels index more synaptic injury). **C** CSF YKL-40 (higher levels index more glial activation). Simple linear correlation analyses: *R* square and *P* value were obtained by fitting a simple linear regression model. Only those analytes associated with either CSF Aβ_42_ or CSF p-tau_181_ in multiple regression analyses are shown

Multiple regression analyses indicated that associations with amyloid pathology (inversely indexed by CSF Aβ_42_) included *APOE4* carrier status (*P* < 0.029), larger total brain ventricle volume (*P* < 0.021), less synaptic injury (Ng, *P* < 0.001), less tau (p-tau_181_, *P* = 0.005), and showed a trend for an association with less glial activation (YLK-40, *P* = 0.097). In simple linear correlation analyses in the overall population, amyloid pathology showed significant inverse correlations of weak to moderate strength with tau pathophysiology (*P* = 0.004), synaptic injury (*P* < 0.001), and glial activation (*P* = 0.028) (Figs. 2 and 3). In *APOE4* and *BCHE-K* carriers*,* inverse correlations were moderate between amyloid pathology and tau pathophysiology (*P* < 0.022) (Fig. 2) and between amyloid pathology and synaptic injury (*P* < 0.001) (Fig. 3B). In *APOE4* carriers without *BCHE-K*, inverse correlations between amyloid with tau pathophysiology were weak (Fig. 2; *P* = 0.042), and between amyloid and synaptic injury or glial activation were moderate (*P* < 0.001 and *P* = 0.013, respectively) (Fig. 3B, C).

### Tau pathophysiology correlated with synaptic injury and glial activation, especially in *APOE4* and *BCHE-K* noncarriers

Multiple regression analyses indicated positive associations between tau pathophysiology (CSF p-tau_181_) and CSF levels of NfL (*P* = 0.002), Ng (*P* < 0.001), and YKL-40 (*P* = 0.01). Thus, more tau pathophysiology associated with increased neuroaxonal damage, synaptic injury, and glial activation. In simple linear regressions in the overall population, tau pathophysiology showed a moderate correlation with neuroaxonal damage (*P* < 0.001), a moderately strong correlation with synaptic injury (*P* < 0.001), and a weak correlation with glial activation (*P* = 0.004) (Fig. 3A-C). In subgroups, correlations of tau pathophysiology with synaptic injury were moderate in *APOE4* carriers without *BCHE-K* (*P* < 0.001), moderately strong in carriers of both *APOE4* and *BCHE-K* (*P* < 0.001), and strong in *APOE4* noncarriers with (*P* = 0.001) and without *BCHE-K* (*P* = 0.003) (Fig. 3B). The correlation between tau pathophysiology and neuroaxonal damage was moderate in the overall group population, but moderately strong in *APOE4* carriers without *BCHE-K* (*P* < 0.001) (Fig. 3A), whereas glial activation was weak in the overall population and in *APOE4* carriers without *BCHE-K* (*P* = 0.043), but was strong in noncarriers of both *APOE4* and *BCHE-K* (*P* = 0.005) (Fig. 3C). The glial activation marker, YKL-40, has been proposed as an indicator of tau pathology [44], and this may be most applicable in *APOE4* and *BCHE-K* noncarriers. In addition, there was a weak positive correlation between glial activation and synaptic injury in *BCHE-K* noncarriers, but strong in *APOE4* noncarriers without *BCHE-K* (*P* = 0.033) and weak in *APOE4* carriers without *BCHE-K* (*P* = 0.029) (Fig. S1). Correlations were absent in *BCHE-K* carriers with or without *APOE4*.

## Discussion

In this sample of clinically and pathologically characterized patients with early AD aged less than 75 years, *APOE4* carriers with *BCHE-K* had a mean age-at-diagnosis of AD 6.4 years earlier than in *APOE4* carriers without *BCHE-K* (Fig. 1A). In *APOE4* carriers, higher accumulations of amyloid and tau pathophysiology were present in *BCHE-K* carriers over 6 years earlier than in *BCHE-K* noncarriers (Table 2). *APOE4* allele frequency-dependent effects on the risk-of-onset of AD are highest in the seventh decade, wane over 70 years of age, and are particularly reduced after 80 years of age [45, 46]. Therefore, the magnitude of the observed modifier effect of *BCHE-K* may be influenced by the younger population age range where the effects of *APOE4* on the AD phenotype are maximal and modifiable. The restricted age range for entry into the current study may also have been at least partly responsible for the lack of any significant *APOE4* allele frequency-dependent effects on age-at-diagnosis of AD or age-at-baseline.

### Amyloid pathology accumulates at an earlier age in carriers of *APOE4* and *BCHE-K*

Amyloid accumulation starts at an earlier age in *APOE4/E4* individuals, followed by *APOE3/E4*, *APOE2/E4*, *APOE3/E3*, and *APOE2/E3 *[13]. The age at which an individual reaches a threshold level of fibrillar Aβ accumulation may correlate with the age of symptom onset [14]. In the current study, amyloid accumulation reached higher levels in genotype groups with lower levels of glial activation, and an inverse correlation was observed between amyloid pathology and glial activation (*P* = 0.028) (Table 2; Fig. 3C). Levels of amyloid pathology across genotype subgroups are compatible with *APOE4* allele frequency-dependent accumulation beginning at an earlier age with the earliest start in *APOE4* carriers with *BCHE-K* alleles, particularly in *APOE4* homozygotes with *BCHE-K* (Table 2).

### Aβ, ApoE, and BuChE modulation of cholinergic signaling changes levels of glial activation across the AD severity stage continuum

Optimally, glial activation may balance the need to attenuate amyloid accumulation and limit the spread of tau pathology [47, 48]. This is an important determinant of the pathology mix and clinical features of early AD, including the age-of-onset of AD, and it links amyloid and tau pathology, cholinergic signaling, and glial activation [33].

The key hypothesis underlying the evolution of AD pathology in carriers of *APOE4* and *BCHE-K* is that, in preclinical AD, activation of glia is net hypofunctional and results in the accumulation of amyloid pathology; in early AD, glial activation and tau and neurodegenerative pathology show focal increases in the MTL; and in later stage disease, glial activation accompanied by tau and neurodegenerative pathology spread across the neocortex. The cholinergic system plays a key role in controlling glial reactivity and function through α7-nAChRs with both rapid focal synaptic signaling and slow diffuse extracellular signaling [6]. In the prodromal stages of AD there is minimal loss of cholinergic neurons but cholinergic dysfunction is apparent [49], whereas in the advanced stages of AD, a severe loss of cortical cholinergic innervation is evident [50]. Thus, cholinergic signaling may be a critical contributor to the evolution of AD and especially in carriers of *APOE4* and *BCHE-K*. ApoE forms soluble and highly stable complexes with cholinesterase enzymes and Aβ, that can oscillate between slow and ultrafast ACh hydrolysis, depending on Aβ availability [29]. Reduced cholinesterase activity and decreased glial activation have been observed in *APOE4* carriers, particularly in individuals with polymorphic variants of genes encoding cholinesterase enzymes with lower activity, such as *BCHE-K *[24, 29]. In a concentration- and aggregation-dependent manner, Aβ signals through α7-nAChRs and influences the extracellular fluid equilibrium between the breakdown of ACh via effects on ACh-hydrolyzing capacity of cholinesterase [22] and the synthesis of ACh via effects on choline acetyltransferase (ChAT) activity [51]. In AD, α7-nAChR expression on astrocytes is positively correlated with neuritic plaque burden [52].

Importantly, the combination of *APOE4*, *BCHE-K,* and Aβ target the cholinergic system to eventually reduce cholinergic signaling. The decreased cholesterol delivery by ApoE4 to the long and extensively arborized axons of the metabolically taxed cholinergic neurons requires them to expend energy on cholesterol synthesis and to consume acetyl coenzyme A (acetyl-CoA) [53]. Acetyl-CoA is an essential substrate for the synthesis of both ACh and lipids that are required for myelin formation and maintenance of cellular membranes [54]. The ascending white matter projections of the basal forebrain cholinergic system may be particularly vulnerable to the combination of Aβ pathology and ApoE4 [55–57]. In both aging and AD, the intraneuronal accumulation of oligomeric assemblies of Aβ_42_ is a relatively selective trait of basal forebrain cholinergic neurons [58, 59]. Endocytic internalization of Aβ-nAChR complexes may underlie intracellular accumulation of Aβ_42_ and the neurotoxic consequences, such as tau phosphorylation [60]. In addition, in a concentration- and aggregation-dependent manner, Aβ targets cholinergic synapses [57]. In an amyloid mouse model, loss of α7-nAChRs reduced Aβ_42_ plaque load, increased soluble Aβ_42_ oligomers, exacerbated learning and memory deficits, and decreased the functionality of the basal forebrain cholinergic system [61]. Thus, α7-nAChRs may be involved in the formation of Aβ plaque, which may represent a glial strategy to prevent the accumulation of synaptotoxic soluble Aβ [62]. In addition, cholinergic stimulation of α7-nAChRs inhibits high-mobility group box 1 (HMGB1) release and activation of the NF-κB pathway; decreased cholinergic activity is associated with increased HMGB1 levels [63]. Neuronal HMGB1 release may be a key mechanism underlying neuronal *APOE4*-driven tau pathology and neurodegeneration [64].

Thus, Aβ, ApoE, and BuChE have physiological and disease roles in both the tuning of cholinergic activity and in the vulnerability of the basal forebrain cholinergic system towards degeneration; these actions influence the functional status of cholinoceptive neuronal and non-excitable cells [17]. Initially, increased cholinergic signaling and hypofunctional glia contribute to the accumulation of amyloid pathology and may be net neuroprotective, but later in the disease, deficient cholinergic signaling and overactivated glia contribute to the spread of tau and synaptic pathology. Moreover, there is reduced cholinergic synaptic and extracellular signaling in both normal and pathological aging that will likely lessen ACh-mediated suppression of glial activation and result in age-related increases in glial activation [65].

### An age- and genotype-dependent phenotypic extreme of limbic-amnestic and amyloid-predominant early AD is associated with low levels of glial activation (Figs. 3 and 4; Table 2)

**Fig. 4:**
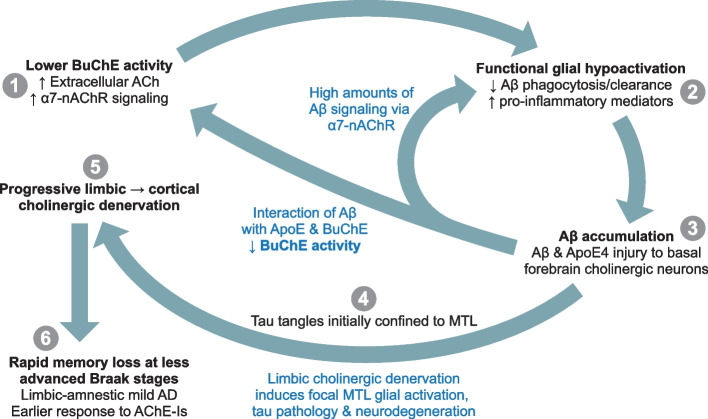
Amyloid pathology facilitating phenotype of early AD exemplified by *APOE4* homozygotes with *BCHE-K* aged < 75 years. In preclinical and prodromal AD, especially in *APOE4* homozygote *BCHE-K* carriers below 75 years of age, lower BuChE activity results in higher extracellular ACh and increased signaling through nAChR on glial cells (1). “*Functionally underactive*” glia with decreased phagocytic, degradative functions, and homeostatic responsiveness (2), impair Aβ clearance resulting in earlier and greater amyloid accumulation (3). In early AD, limbic cholinergic denervation due to ApoE4, Aβ, and tau-mediated damage to basal forebrain cholinergic neurons that project to corticolimbic regions, removes the cholinergic “brake” on glia to increase glial activation, tau pathology and neurodegeneration in the MTL (4). The spread of this pathology outside of the MTL is initially limited until cholinergic denervation has progressed to include other neocortical areas (5). Thus, high levels of amyloid accumulation develop at a younger age, and basal forebrain cholinergic denervation of MTL structures results in hippocampal atrophy and a rapidly progressing limbic-amnestic presentation in early AD with good response to AChE-Is (6). The progression of corticolimbic cholinergic denervation to neocortical areas beyond the MTL results in the spread of glial activation, tau pathology, and neurodegeneration

At one end of the age-, genotype-, and disease stage-dependent spectrum of early AD is the limbic-amnestic and amyloid accumulation-predominant phenotype, exemplified by *APOE4* homozygotes with *BCHE-K* alleles who are aged less than 75 years (Table 2). In *APOE4* carriers with *BCHE-K*, greater accumulation of Aβ pathology at younger ages is likely due to greater deficits in glial-mediated clearance mechanisms [7, 66] (Figs. 3C and 4). These functionally underactive glia may produce proinflammatory cytokines and be classified as “inflammatory,” but their endolysosomal and phagocytic functions may be greatly reduced [67]. Putatively lower BuChE activity in *BCHE-K* carriers with *APOE4* alleles results in higher extracellular ACh, further reduces the phagocytic function and responsiveness of glial cells, and further impairs Aβ clearance. While excessive cholinergic signaling encourages high levels of amyloid accumulation, the spread and accumulation of tau pathology are simultaneously kept in check (Figs. 2 and 4). However, in early AD, *APOE4* is associated with rapidly increasing tau pathology as the combination of ApoE4 and Aβ pathology induces sufficient degeneration of basal forebrain corticolimbic cholinergic neurons to release the cholinergic “brake” on glial activation in the medial temporal lobe (MTL) and eventually in other cortical regions (Fig. 4). Cholinergic denervation and tau pathology accumulating in the MTL result in a rapidly progressing limbic-amnestic presentation with a response to AChE-I that is apparent in the early stages of AD [32, 33]. The focal MTL/limbic denervation is indexed by a more limbic-amnestic presentation (memory > visuospatial impairment, and hippocampal atrophy > ventricular expansion) (Table 2). Although pre-synaptic cholinergic denervation of the MTL induces glial activation, the consequent focal pathology in this region is not fully reflected in CSF measures of glial activation, tau, and neurodegenerative pathology as CSF assessments summarize pathology across the brain. However, these CSF indices will likely show rapid increases as AD progresses and these pathologies spread across the neocortex.

In the limbic-amnestic phenotype, higher amyloid accumulation and lower levels of glial activation were more prominent in *APOE4* carriers (Fig. 2). The limbic-amnestic deficits are likely caused by greater and earlier degeneration of the basal forebrain corticolimbic cholinergic projection system that is the result of deficient glial clearance of Aβ. In early-stage AD, *APOE4* homozygotes with *BCHE-K* had the lowest levels of glial activation, neurodegeneration, and tau pathophysiology (Table 2), whereas indices of these pathologies were presumably higher in *APOEɛ4* heterozygotes with *BCHE-K*. In *APOEɛ4* heterozygotes the presence of an *APOE3* allele (or a rare *APOE2* allele) allows for higher levels of glial activation and spread of tau and neurodegenerative pathology. In *APOE4* noncarriers without *BCHE-K* there is less restraint on glial activation exhibited by a strong correlation between glial activation and tau pathophysiology *(R*^*2*^ = 0.825, *P* = 0.005).

Thus, in *APOE4* homozygotes with *BCHE-K* with mild AD below the age of 75 years, increases in tau pathology first appear in the MTL and these increases are dependent on removal or denervation of the corticolimbic cholinergic “brake” on glial activation. In early AD, *APOE4* homozygotes with *BCHE-K* still have the lowest levels of tau and neurodegenerative pathology, and are on a slightly different journey to end-stage disease than *APOE4* heterozygotes with *BCHE-K*. *APOE4* homozygotes with *BCHE-K* evolve a rapidly increasing burden of tau and neurodegenerative pathology as they progress along the severity continuum toward end-stage disease, where they will have similar levels of pathology to other genotype groups [12].

### Hypofunctional glial-mediated clearance of Aβ likely underlies the amyloid accumulation in carriers of *APOE4* and *BCHE-K* aged < 75 years

Balanced glil activation may be needed to stimulate Aβ clearance and avoid the two extremes of “hypofunctional” glia that promote amyloid accumulation and “hyperactivated” glia that facilitate the dissemination of tau [47, 48, 68]. In amyloid mouse models, inhibition of reactive astrogliosis increases Aβ_42_ plaque burden [69], whereas shifting microglia to an interferon-responsive state boosts ApoE expression, phagocytosis, containment of plaques, and lessens damage to nearby neurons and synapses [70]. However, further shifting of microglia to an overactivated state may increase synaptic engulfment and accelerate the dissemination of tau pathology [71]. Mouse models indicate that ApoE controls glial activation, but ApoE4 locks microglia in a homeostatic state, decreasing in phagocytic capacity, and resulting in a failure to clear pathological debris [7, 66, 72]. Therefore, microglial and astrocyte coverage of plaques is likely protective for surrounding neurons, and ApoE4 is associated with decreased coverage and more neuronal dystrophy [73–75].

*BCHE-K* carriers, who have lower levels of glial activation markers and higher levels of proinflammatory cytokines [24, 29], may exhibit deficient glial responses to neurodegeneration. Rapid and appropriately tuned changes in the catalytic activity of BuChE, and the necessary adaptive changes in cholinoceptive non-excitable cells, may be more difficult to achieve with BuChE-K, particularly in *APOE4* carriers. Carriers of *APOE4* and *BCHE-K* may have highly senescent microglia that are associated with blocked endolysosomal processing, impaired phagocytosis, and accelerated Aβ_42_ pathology [76]. YKL-40 is a context-dependent indicator of glial phagocytic activity in both mice and humans [77]. In the current study, *APOE4* noncarriers had higher mean levels of CSF YKL-40 (318 ± 162 ng/mL), relative to *APOE4* carriers (247 ± 106 ng/mL; (data not shown, [43]). Mean levels were further reduced in *APOE4* homozygotes (202 ± 64 ng/mL) and in *APOE4* homozygotes with *BCHE-K* alleles (190 ± 86 ng/mL; Table 2). This likely explains the inverse correlations observed between amyloid pathology and YKL-40, and between amyloid pathology and synaptic injury in the overall population and in *APOE4* carriers; these correlations were absent in *APOE4* non-carriers (Fig. 3B and C).

### Examination at younger ages may elucidate hypofunctional glial-mediated clearance of Aβ in carriers of *APOE4* and *BCHE-K*

Increases in functional glial activation with age might explain why there is a decreased *APOE4*-associated risk for AD from of 70–80 years, and why progression to dementia in carriers of both *APOE4* and *BCHE-K* is at least 2-fold greater below 75 years of age compared to older carriers [33]. In a longitudinal study of prodromal AD, 39% of *APOE4* and *BCHE-K* carriers aged less than 75 years progressed to AD over 3–4 years, while 18% of patients aged 75 years or more progressed to AD over 3–4 years [33]. This contrasted with the overall study population, where progression to AD was greater in older patients (29%), compared to those aged less than 75 years (13%). Younger *APOE4* carriers have accelerated progression of hippocampal atrophy in prodromal and early-stage AD, but in individuals who are more advanced in age or progression of disease, the influence of *APOE4* on hippocampal atrophy is lost [78]. Conversely, global cerebral atrophy in AD patients with a mean age of 70 years was reduced in an *APOE4* allele frequency-dependent manner [79], whereas in older patients, with a mean age of 80 years, atrophy was not different by genotype [78, 80]. Thus, the findings in the current study are likely age-dependent and should not be extrapolated to early AD patients aged over 75 years.

### The “amyloid accumulating and initially tau spread limiting” phenotype does not contradict the amyloid cascade hypothesis

In a previous study, carriers of *APOE4* and *BCHE-K* with prodromal AD exhibited disease progression rates inversely correlated with age and hippocampal volume, and showed the greatest decline in short- and long-term retrieval from verbal memory and in overall cognitive impairment [27, 33]. Likewise, in the current study of patients with early AD, *APOE4* carriers had greater memory deficits, hippocampal atrophy, and amyloid accumulation relative to noncarriers. Additionally, these pathologies occurred ~ 6 years earlier in *APOE4* carriers with *BCHE-K* compared to *APOE4* carriers without *BCHE-K* (Fig. 1C, Table 2). The amyloid cascade hypothesis of AD implies that reaching the threshold for parenchymal amyloid positivity at an earlier age should drive secondary effector tau pathology, with an earlier age-at-onset of AD [81]. Support for this hypothesis comes from slower progression of tau tangle accumulation and slower cognitive decline following antibody-induced removal of amyloid plaque to below key thresholds in patients with early AD [82, 83]. However, in the current study, correlations of amyloid pathology with tau pathophysiology in *APOE4* carriers were negative, particularly in carriers of both *APOE4* and *BCHE-K* (Fig. 2). Across groups defined by *APOE4* and *BCHE-K* carrier status, carriers of both alleles had the highest amyloid pathology (Table 2). Amyloid pathology was further increased in *APOE4* homozygotes with *BCHE-K*, and accompanied by the lowest levels of tau and neurodegenerative pathology (Table 2).

The inverse correlations of amyloid pathology with tau pathophysiology and synaptic injury may be a consequence of tau and neurodegenerative pathology localized to the MTL (which includes the entorhinal cortex, amygdala, and hippocampus). This MTL tau and neurodegenerative pathology may be responsible for transitioning *APOE4* and *BCHE-K* carriers into AD at an earlier age with less global tau and neurodegenerative pathology (Table 2; Fig. 4) [84, 85]. Such neuroanatomical distinctions are not discernible from CSF assessments that summarize pathology changes across the brain. Spatial resolution requires tau tangle-ligand positron-emission tomography (tau-PET) neuroimaging. In the presence of global amyloid pathology in *APOE4* carriers, tau-PET indicates that tau pathology is more severe with a focal MTL distribution [85, 86]. Furthermore, younger age is associated with a PET-tau signal in the MTL of early AD *APOE4* carriers, but this is not seen in *APOE4* noncarriers [85]. Across the aging and AD spectrum, *APOE4* carriers present with increased microglial activation relative to noncarriers in early Braak stage regions within the MTL. This microglial activation mediates Aβ-independent effects of *APOE4* on tau accumulation that are further associated with neurodegeneration and clinical impairment [87].

Degeneration of basal forebrain cholinergic neurons that project to the MTL and other cortical structures precedes and predicts longitudinal entorhinal/MTL degeneration [88, 89]. Notably, preclinical *APOE4* carriers exhibit the greatest loss of basal forebrain volume [90]. The ascending corticolimbic neuronal projections of the basal forebrain cholinergic system may be particularly vulnerable to the combination of ApoE4-mediated glial hypofunction and impaired lipid dynamics, and high levels of Aβ and pathological tau [55–57]. The impact of focal basal forebrain pathology is magnified as it causes widespread presynaptic cholinergic corticolimbic denervation. Both amyloid and tau pathologies may be required for substantial impairment of cholinergic synaptic plasticity and memory, and for continuous destruction of the projecting branches of the cholinergic nuclei in the basal forebrain [91, 92].

### Implications for future clinical research and development of therapeutics

Findings in the current study, if confirmed, could have implications for the conceptualization of Alzheimer pathological cascades, identification of therapeutic targets, and usage of existing and future treatments. The genetic architecture of prognosis in AD is fundamental for proper medical care and in the design and interpretation of clinical trials. Despite its importance, the genetic architecture of prognosis is less established than the genetics of susceptibility. *BCHE-K* may join *APOE4* allele frequency, age, and sex as a foundational component of predictive modeling for early AD phenotypes [93, 94]. Moreover, the cholinergic hypothesis appears seamlessly interlinked with the amyloid cascade hypothesis. *APOE* and *BCHE* genotypes appear to exert a critical influence on the functional activation of glia as indexed by the microglial and astrocyte activation CSF marker, YKL-40. The level of extracellular cholinergic signaling to cholinoceptive cells, including glia, depends on the enzymatic activity of BuChE, which is dependent on BuChE levels, *BCHE* polymorphic variation, ApoE levels, *APOE* polymorphic variation, and soluble Aβ levels. The health of cholinergic neurotransmission and extracellular signaling systems may be crucial to healthy brain aging [95].

The quantitative removal of amyloid pathology with anti-amyloid antibodies is dependent on Fc receptor-mediated phagocytosis and clearance of Aβ [82, 83]. Response to this targeted immune-activating therapeutic approach may vary depending on the individual’s predominant microglial state. While some beneficial effects might be caused by antibody-mediated stimulation of glia with improved performance of homeostatic functions [96], stimulating the clearance of Aβ prior to substantial levels of corticolimbic cholinergic denervation and spread of tau pathology may produce the best outcomes in *APOE4* carriers below the age of 75 years. However, this may require intervention in asymptomatic individuals. In addition, longer treatment durations may be necessary in substantial amyloid accumulators, such as *APOE4* homozygotes with *BCHE-K* alleles, to push amyloid levels below the threshold and to prevent or slow further corticolimbic cholinergic denervation and tau pathology.

While a retuning of innate immune responses may be required to harness protective and beneficial effects and to attenuate negative effects, the required changes will differ across a genotype, age, and the disease stage continuum. Tuning in the wrong direction will simply make matters worse. Considerations may be further complicated by the nuances and complexity of glial cell phenotypes across different brain regions, between adjacent glia, and in different disease contexts [97]. The challenge in the development of potential amyloid pathology limiting therapeutics may not lie in simply upregulating the activation state of glia.

## Limitations

Strengths of the study include the well-characterized sample of individuals aged less than 75 years with CSF biomarkers and standardized clinical assessments in expert clinical settings. The limitations of this investigation include its small size, cross-sectional design, and the possibly unrepresentative nature of those enrolled in an interventional clinical trial. Moreover, inferences from the data were based on associations at a particular point in time (baseline) that cannot evidence causal effects, and many correlations involved small sample sizes. Evaluation of the age-at-diagnosis of AD might have benefited from the use of standardized prospective assessments of diagnosis and of onset-age across study sites. Nonetheless, similar genotype group relationships were also demonstrated on the age-at-baseline of study, i.e., at the time this cross-sectional investigation was conducted. Prospective longitudinal assessment in larger samples is necessary to better evaluate phenotypic evolution along the AD continuum and to confirm and develop these findings.

Additionally, more extensive mapping of inflammatory mediators, complement and myelin markers, and CSF ApoE and BChE levels and activity may be illuminating. The primary biomarker used in this study to index tau pathophysiology (CSF p-tau_181_) may reflect a mix of amyloid and tau pathological changes in the brain [98], and is therefore not a “pure” marker of tau tangle load in the brain. Glia were simplistically ascribed activated or hypofunctional phenotypes, and as facilitating or limiting amyloid accumulation or tau spread. The association of levels of the astrocyte and microglial activation marker, CSF YKL-40, with transcriptional, morphological, and functional states of glia are not clear, and large multiomic datasets and machine learning may be required to elucidate them.

The majority of study participants were of European ancestry; heterogeneity in the genetic neighborhood of these genes and local *APOE* and *BCHE* haplotypes may be of importance when interpreting these results [99]. In addition, other genetic variants of *BCHE* were not assessed in the current study [100], and some have been shown to unequivocally impact the amyloid cascade [101]. Lastly, discerning clinical phenotypes in different genotype subgroups on a variable background of ChE-I therapy and medications with potential anticholinergic properties may be problematic, as ChE-I therapy can influence phenotypic expression. For example, *APOE4* carriers—especially those with concomitant *BCHE-K* alleles—are particularly responsive to AChE-I treatment in the mild stage of AD, and the magnitude of attention, processing speed, and amnestic deficits in these individuals may have been partly obscured [32].

## Conclusion

Below the age of 75 years, AD may be more monocausal, without substantial contributions from other age-related pathologies, and the influence of modifying genetic variation on the phenotype of early AD may be more apparent. In *APOE4* carriers, the presence versus the absence of *BCHE-K* alleles associated with a significantly earlier mean age-at-diagnosis of AD of 6.4 years, a more limbic-amnestic phenotype, and similar accumulations of amyloid and tau pathology but more than 6 years earlier. Thus, in *APOE4* carriers below the age of 75 years, a major contribution to earlier age-at-diagnosis of AD may be concomitant *BCHE-K* alleles. In *APOE4* carriers, *BCHE-K* further reduces the functional activation of glia by increasing cholinergic synaptic signaling from basal forebrain corticolimbic cholinergic neuronal projections and extracellular cholinergic signaling through cell surface α7-nAChRs. However, the further lowering of glial activation results in earlier amyloid pathology accumulation that, in combination with ApoE4, is particularly damaging to basal forebrain corticolimbic cholinergic neurons. The spread of tau and synaptic pathology from the MTL to other cortical areas parallels the denervation of corticolimbic cholinergic projections, removal of the cholinergic “brake” on cortical glial activation, and the onset and progression of symptoms. The functional activation of glia, the amyloid cascade hypothesis, and the cholinergic hypothesis of AD are interwoven. In this early AD population, the concept has the potential to explain much of the phenotypic heterogeneity and to enable more appropriate use of existing, emerging, and future therapies. Confirmation of these post hoc findings in larger, prospective, and longitudinal studies is required.

### Supplementary Information

**Additional file 1:** Raw data (including Mean [SD, SEM], Median [P25, P75], and Min/Max). **Table S1.** Early AD phenotype across genotype groups defined by *BCHE-K *allele frequency. **Table S2.** Early AD phenotype across genotype groups defined by *APOE4 *and *BCHE-K* carrier status.

**Additional file 2.** Ethics committees approving clinical study.

**Additional file 3: Figure S1.** Correlations in the overall population and in *APOE4* and *BCHE-K *subgroups of Ng versus YKL-40.

## Data Availability

No datasets were generated or analysed during the current study.
