# Supplementary material for: Onset of Alzheimer disease in apolipoprotein ɛ4 carriers is earlier in butyrylcholinesterase K variant carriers
Source: BMC Neurol. 2024 Apr 9;24:116. doi: 10.1186/s12883-024-03611-5 (PMC11003149; doi:10.1186/s12883-024-03611-5)
Supplement: Supplementary file 1 — Additional file 1: Raw data (including Mean [SD, SEM], Median [P25, P75], and Min/Max). Table S1. Early AD phenotype across genotype groups defined by BCHE-K allele frequency. Table S2. Early AD phenotype across genotype groups defined by APOE4 and BCHE-K carrier status. [file 12883_2024_3611_MOESM1_ESM.docx]

## Table S1. Mild AD phenotype across genotype groups defined by *BCHE-K allele* frequency.

| Variable |  |  | *BCHE-K* carrier  (n=16) | 2 *BCHE-K*  (n=3) | 1 *BCHE-K*  (n=13) | No *BCHE-K*  (n=29) |
| --- | --- | --- | --- | --- | --- | --- |
| Sex | Female |  | 9  (56.3%) | 2  (66.7%) | 7  (53.8%) | 13  (44.8%) |
|  | Male |  | 7  (43.8%) | 1  (33.3%) | 6  (46.2%) | 16  (55.2%) |
| Onset of AD and age-at-baseline | Age-at-diagnosis  (yrs) | **Mean**  **(SD, SEM)** | **61.63**  **(6.51, 1.63)** | **59.37^1^**  **(5.44, 3.14)** | **62.16^1^**  **(6.82, 1.89)** | **66.02^1^**  **(5.30, 0.98)** |
|  |  | Median  (P25, P75) | 61.58  (56.64, 66.08) | 57.92  (54.80, 65.39) | 61.89  (56.69, 66.33) | 66.48  (63.10, 70.23) |
|  |  | (Min, Max) | (49.04, 74.13) | (54.80, 65.39) | (49.04, 74.13) | (47.99, 72.79) |
|  | Age-at-baseline  (yrs) | **Mean**  **(SD, SEM)** | **62.9**  **(6.2, 1.6)** | **60.0**  **(5.6, 3.2)** | **63.6**  **(6.4, 1.8)** | **67.4**  **(4.9, 0.9)** |
|  |  | Median  (P25, P75) | 63.0  (59.0, 67.5) | 59.0  (55.0, 66.0) | 63.0  (59.0, 68.0) | 67.0  (66.0, 71.0) |
|  |  | (Min, Max) | (50.0, 74.0) | (55.0, 66.0) | (50.0, 74.0) | (52.0, 74.0) |
| Neuroimaging | Hippocampal vol., % of ICV | **Mean**  **(SD, SEM)** | **0.27**  **(0.04, 0.01)** | **0.27**  **(0.02, 0.01)** | **0.27**  **(0.04, 0.01)** | **0.25**  **(0.04, 0.01)** |
|  |  | Median  (P25, P75) | 0.27  (0.26, 0.29) | 0.27  (0.25, 0.29) | 0.27  (0.27, 0.29) | 0.24  (0.22, 0.27) |
|  |  | (Min, Max) | (0.17, 0.33) | (0.25, 0.29) | (0.17, 0.33) | (0.19, 0.34) |
|  | Ventricular vol., % of ICV | **Mean**  **(SD, SEM)** | **2.72**  **(1.38, 0.35)** | **1.84**  **(0.76, 0.44)** | **2.93**  **(1.43, 0.40)** | **2.82**  **(1.19, 0.22)** |
|  |  | Median  (P25, P75) | 2.38  (1.81, 3.58) | 1.75  (1.12, 2.65) | 2.80  (2.00, 3.86) | 2.67  (1.94, 3.49) |
|  |  | (Min, Max) | (1.07, 6.20) | (1.12, 2.65) | (1.07, 6.20) | (1.02, 5.11) |
| CSF markers | Aβ_42_, pg/mL | **Mean**  **(SD, SEM)** | **676.1**  **(160.9, 40.2)** | **657.3**  **(220.2, 127.1)** | **680.5**  **(155.5, 43.1)** | **713.2**  **(198.1, 36.8)** |
|  |  | Median  (P25, P75) | 653.5  (570.1, 805.0) | 675.8  (428.5, 867.7) | 642.3  (572.0, 804.8) | 687.1  (592.2, 864.6) |
|  |  | (Min, Max) | (428.5, 1039.0) | (428.5, 867.7) | (491.4, 1039.0) | (340.4, 1126.0) |
|  | p-tau_181_, pg/mL | **Mean**  **(SD, SEM)** | **41.06**  **(12.91, 3.23)** | **41.95**  **(13.93, 8.05)** | **40.85**  **(13.25, 3.68)** | **39.11**  **(14.30, 2.65)** |
|  |  | Median  (P25, P75) | 38.96  (31.52, 51.20) | 36.07  (31.91, 57.86) | 39.36  (31.12, 48.31) | 35.57  (30.29, 44.83) |
|  |  | (Min, Max) | (19.71, 63.98) | (31.91, 57.86) | (19.71, 63.98) | (20.55, 81.28) |
|  | NfL, pg/mL | **Mean**  **(SD, SEM)** | **1368.151**  **(416.737, 104.184)** | **1250.411**  **(270.156, 155.974)** | **1395.322**  **(447.949, 124.239)** | **1279.087**  **(395.142, 73.376)** |
|  |  | Median  (P25, P75) | 1270.970  (1139.410, 1538.598) | 1213.900  (1000.368, 1536.965) | 1296.260  (1167.610, 1540.230) | 1325.795  (999.923, 1564.960) |
|  |  | (Min, Max) | (630.833, 2391.810) | (1000.368, 1536.965) | (630.833, 2391.810) | (580.969, 2223.485) |
|  | Ng, pg/mL | **Mean**  **(SD, SEM)** | **559.330**  **(199.224, 49.806)** | **560.000**  **(238.627, 137.771)** | **559.176**  **(200.305, 55.555)** | **508.231**  **(250.610, 46.537)** |
|  |  | Median  (P25, P75) | 541.329  (392.145, 744.592) | 538.994  (332.570, 808.435) | 543.664  (422.006, 738.869) | 470.174  (315.335, 562.083) |
|  |  | (Min, Max) | (237.776, 905.365) | (332.570, 808.435) | (237.776, 905.365) | (194.618, 1306.915) |
|  | YKL-40, pg/mL | **Mean**  **(SD, SEM)** | **279704.0**  **(152934.4, 38233.6)** | **226656.3**  **(75313.2, 43482.1)** | **291945.8**  **(165604.8, 45930.5)** | **258422.6**  **(109116.7, 20262.5)** |
|  |  | Median  (P25, P75) | 230043.5  (147203.5, 382781.8) | 239378.0  (145792.5, 294798.5) | 220709.0  (148614.5, 385381.5) | 236943.0  (202933.5, 300130.5) |
|  |  | (Min, Max) | (98750.8, 598333.5) | (145792.5, 294798.5) | (98750.8, 598333.5) | (112021.5, 662666.5) |
| Cognition | MMSE Total  (0-30) | **Mean**  **(SD, SEM)** | **23.7**  **(2.4, 0.6)** | **22.3**  **(1.5, 0.9)** | **24.0**  **(2.5, 0.7)** | **23.6**  **(2.2, 0.4)** |
|  |  | Median  (P25, P75) | 24.5  (21.5, 26.0) | 22.0  (21.0, 24.0) | 25.0  (22.0, 26.0) | 23.0  (22.0, 26.0) |
|  |  | (Min, Max) | (20.0, 27.0) | (21.0, 24.0) | (20.0, 27.0) | (20.0, 27.0) |
|  | MMSE Memory  (0-6) | **Mean**  **(SD, SEM)** | **4.0**  **(1.1, 0.3)** | **3.3**  **(0.6, 0.3)** | **4.2**  **(1.1, 0.3)** | **4.3**  **(1.1, 0.2)** |
|  |  | Median  (P25, P75) | 4.0  (3.0, 4.5) | 3.0  (3.0, 4.0) | 4.0  (4.0, 5.0) | 4.0  (3.0, 5.0) |
|  |  | (Min, Max) | (2.0, 6.0) | (3.0, 4.0) | (2.0, 6.0) | (3.0, 6.0) |
|  | MMSE Visual Construction  (0-1) | **Mean**  **(SD, SEM)** | **0.8**  **(0.4, 0.1)** | **0.7**  **(0.6, 0.3)** | **0.8**  **(0.4, 0.1)** | **0.6**  **(0.5, 0.1)** |
|  |  | Median  (P25, P75) | 1.0  (0.5, 1.0) | 1.0  (0.0, 1.0) | 1.0  (1.0, 1.0) | 1.0  (0.0, 1.0) |
|  |  | (Min, Max) | (0.0, 1.0) | (0.0, 1.0) | (0.0, 1.0) | (0.0, 1.0) |
|  | RBANS Total  (40-160) | **Mean**  **(SD, SEM)** | **67.5**  **(11.5, 2.9)** | **63.3**  **(15.6, 9.0)** | **68.5**  **(10.9, 3.0)** | **67.1**  **(12.0, 2.2)** |
|  |  | Median  (P25, P75) | 71.5  (56.0, 77.0) | 61.0  (49.0, 80.0) | 72.0  (57.0, 77.0) | 66.0  (59.0, 76.0) |
|  |  | (Min, Max) | (49.0, 82.0) | (49.0, 80.0) | (51.0, 82.0) | (49.0, 90.0) |
|  | RBANS Delayed Memory  (40-154) | **Mean**  **(SD, SEM)** | **57.8**  **(19.2, 4.8)** | **58.7**  **(22.7, 13.1)** | **57.6**  **(19.4, 5.4)** | **51.8**  **(13.3, 2.5)** |
|  |  | Median  (P25, P75) | 50.0  (44.0, 66.0) | 52.0  (40.0, 84.0) | 48.0  (44.0, 64.0) | 48.0  (44.0, 52.0) |
|  |  | (Min, Max) | (40.0, 102.0) | (40.0, 84.0) | (40.0, 102.0) | (40.0, 94.0) |
|  | RBANS Visuospatial/ Constructional  (40-154) | **Mean**  **(SD, SEM)** | **86.9**  **(24.1, 6.0)** | **79.0**  **(36.4, 21.0)** | **88.8**  **(22.1, 6.1)** | **84.2**  **(20.5, 3.8)** |
|  |  | Median  (P25, P75) | 78.0  (64.0, 105.0) | 60.0  (56.0, 121.0) | 78.0  (72.0, 105.0) | 84.0  (64.0, 102.0) |
|  |  | (Min, Max) | (56.0, 126.0) | (56.0, 121.0) | (62.0, 126.0) | (50.0, 121.0) |

**^1^** = Significant difference across groups, (*P* = .048, ANOVA); ICV, intracranial volume; MMSE, Mini-Mental Status Examination; RBANS, Repeatable Battery for the Assessment of Neuropsychological Status; SD, standard deviation; SEM, standard error of the mean.

## Table S2. Mild AD phenotype across genotype groups defined by *APOE4* and *BCHE-K* carrier status.

| Variable |  |  | *2E4 & K*  (N = 4) | *E4* & *K*  (N = 11) | *E4* & No-*K*  (N = 22) | No-*E4* & *K*  (N = 5) | No-*E4* & No-*K*  (N = 7) |
| --- | --- | --- | --- | --- | --- | --- | --- |
| Sex | Female |  | 3  (75.0%) | 7  (63.6%) | 11  (50.0%) | 2  (40.0%) | 2  (28.6%) |
|  | Male |  | 1  (25.0%) | 4  (36.4%) | 11  (50.0%) | 3  (60.0%) | 5  (71.4%) |
| Onset of AD and age-at-baseline | Age-at-diagnosis  (yrs) | **Mean**  **(SD, SEM)** | **60.77**  **(4.41, 2.20)** | **60.61**  **(6.08, 1.83)** | **67.03**  **(3.96, 0.84)** | **63.88**  **(7.58, 3.39)** | **62.84**  **(7.78, 2.94)** |
|  |  | Median  (P25, P75) | 61.45  (57.90, 63.64) | 61.89  (54.80, 65.83) | 67.16  (64.04, 70.63) | 61.28  (57.92, 69.41) | 63.00  (59.16, 69.21) |
|  |  | (Min, Max) | (54.80, 65.39) | (49.04, 68.44) | (59.37, 72.79) | (56.69, 74.13) | (47.99, 71.45) |
|  | Age-at-baseline  (yrs) | **Mean**  **(SD, SEM)** | **62.0**  **(4.8, 2.4)** | **62.1**  **(5.9, 1.8)** | **68.3**  **(4.0, 0.9)** | **64.8**  **(7.3, 3.2)** | **64.4**  **(6.6, 2.5)** |
|  |  | Median  (P25, P75) | 63.5  (59.0, 65.0) | 63.0  (59.0, 67.0) | 68.0  (66.0, 72.0) | 62.0  (59.0, 71.0) | 66.0  (60.0, 69.0) |
|  |  | (Min, Max) | (55.0, 66.0) | (50.0, 69.0) | (62.0, 74.0) | (58.0, 74.0) | (52.0, 72.0) |
| Neuroimaging | Hippocampal vol., % of ICV | **Mean**  **(SD, SEM)** | **0.26**  **(0.05, 0.03)** | **0.26**  **(0.05, 0.01)** | **0.24**  **(0.03, 0.01)** | **0.28**  **(0.02, 0.01)** | **0.28**  **(0.03, 0.01)** |
|  |  | Median  (P25, P75) | 0.28  (0.23, 0.29) | 0.27  (0.24, 0.29) | 0.24  (0.21, 0.25) | 0.29  (0.27, 0.30) | 0.28  (0.27, 0.30) |
|  |  | (Min, Max) | (0.18, 0.29) | (0.17, 0.33) | (0.19, 0.34) | (0.25, 0.31) | (0.22, 0.32) |
|  | Ventricular vol., % of ICV | **Mean**  **(SD, SEM)** | **1.77**  **(0.73, 0.36)** | **2.91**  **(1.54, 0.46)** | **2.79**  **(1.23, 0.26)** | **2.30**  **(0.97, 0.44)** | **2.91**  **(1.15, 0.44)** |
|  |  | Median  (P25, P75) | 1.66  (1.18, 2.37) | 2.65  (1.86, 3.86) | 2.59  (1.94, 3.61) | 2.12  (1.75, 3.26) | 2.87  (1.93, 3.49) |
|  |  | (Min, Max) | (1.12, 2.65) | (1.12, 6.20) | (1.02, 5.02) | (1.07, 3.31) | (1.92, 5.11) |
| CSF markers | Aβ_42_, pg/mL | **Mean**  **(SD, SEM)** | **553.9**  **(110.6, 55.3)** | **640.5**  **(138.6, 41.8)** | **675.7**  **(193.2, 41.2)** | **754.5**  **(194.7, 87.1)** | **831.2**  **(176.1, 66.6)** |
|  |  | Median  (P25, P75) | 555.7  (464.5, 643.4) | 642.3  (500.4, 804.8) | 661.5  (563.9, 773.6) | 669.3  (628.3, 867.7) | 845.8  (647.7, 917.2) |
|  |  | (Min, Max) | (428.5, 675.8) | (428.5, 849.7) | (340.4, 1059.5) | (568.3, 1039.0) | (598.1, 1126.0) |
|  | p-tau_181_, pg/mL | **Mean**  **(SD, SEM)** | **34.00**  **(11.80, 5.90)** | **40.39**  **(12.21, 3.68)** | **38.71**  **(14.83, 3.16)** | **42.52**  **(15.76, 7.05)** | **40.37**  **(13.46, 5.09)** |
|  |  | Median  (P25, P75) | 33.99  (25.81, 42.19) | 38.56  (31.91, 48.31) | 33.06  (28.28, 50.94) | 46.53  (29.15, 56.05) | 36.99  (30.87, 41.36) |
|  |  | (Min, Max) | (19.71, 48.31) | (19.71, 63.98) | (20.55, 81.28) | (23.03, 57.86) | (28.79, 69.12) |
|  | NfL, pg/mL | **Mean**  **(SD, SEM)** | **1003.178**  **(264.632, 132.316)** | **1391.727**  **(498.369, 150.264)** | **1222.185**  **(394.703, 84.151)** | **1316.283**  **(159.506, 71.333)** | **1457.920**  **(366.323, 138.457)** |
|  |  | Median  (P25, P75) | 1083.989  (815.600, 1190.755) | 1213.900  (1036.835, 1850.245) | 1177.855  (868.289, 1488.770) | 1296.260  (1245.680, 1391.300) | 1440.645  (1042.665, 1709.570) |
|  |  | (Min, Max) | (630.833, 1213.900) | (630.833, 2391.810) | (580.969, 2223.485) | (1111.210, 1536.965) | (999.923, 1992.835) |
|  | Ng, pg/mL | **Mean**  **(SD, SEM)** | **493.087**  **(187.517, 93.759)** | **567.892**  **(188.029, 56.693)** | **506.050**  **(257.391, 54.876)** | **540.495**  **(244.553, 109.367)** | **515.083**  **(247.274, 93.461)** |
|  |  | Median  (P25, P75) | 450.454  (347.242, 638.931) | 538.994  (422.006, 738.869) | 471.601  (307.055, 562.083) | 543.664  (362.283, 750.315) | 440.584  (315.335, 642.066) |
|  |  | (Min, Max) | (332.570, 738.869) | (332.570, 905.365) | (194.618, 1306.915) | (237.776, 808.435) | (302.237, 1013.229) |
|  | YKL-40, pg/mL | **Mean**  **(SD, SEM)** | **190012.7**  **(86035.8, 43017.9)** | **247158.4**  **(143270.3, 43197.6)** | **247263.5**  **(85011.5, 18124.5)** | **351304.4**  **(164534.1, 73581.9)** | **293494.2**  **(168456.1, 63670.4)** |
|  |  | Median  (P25, P75) | 183250.8  (122271.6, 257753.8) | 206109.0  (145792.5, 294798.5) | 239475.3  (197204.5, 300613.0) | 380182.0  (239378.0, 487142.5) | 235319.5  (202933.5, 300130.5) |
|  |  | (Min, Max) | (98750.8, 294798.5) | (98750.8, 598333.5) | (112021.5, 487722.0) | (131158.5, 518661.0) | (174219.0, 662666.5) |
| Cognition | MMSE Total  (0-30) | **Mean**  **(SD, SEM)** | **24.3**  **(2.5, 1.3)** | **23.8**  **(2.5, 0.7)** | **23.5**  **(2.3, 0.5)** | **23.4**  **(2.4, 1.1)** | **23.9**  **(2.3, 0.9)** |
|  |  | Median  (P25, P75) | 24.5  (22.5, 26.0) | 25.0  (21.0, 26.0) | 23.0  (22.0, 26.0) | 22.0  (22.0, 26.0) | 24.0  (21.0, 26.0) |
|  |  | (Min, Max) | (21.0, 27.0) | (20.0, 27.0) | (20.0, 27.0) | (21.0, 26.0) | (21.0, 26.0) |
|  | MMSE Memory  (0-6) | **Mean**  **(SD, SEM)** | **4.0**  **(1.4, 0.7)** | **3.9**  **(1.0, 0.3)** | **4.1**  **(1.1, 0.2)** | **4.2**  **(1.3, 0.6)** | **4.9**  **(1.2, 0.5)** |
|  |  | Median  (P25, P75) | 3.5  (3.0, 5.0) | 4.0  (3.0, 4.0) | 4.0  (3.0, 5.0) | 4.0  (3.0, 5.0) | 5.0  (4.0, 6.0) |
|  |  | (Min, Max) | (3.0, 6.0) | (2.0, 6.0) | (3.0, 6.0) | (3.0, 6.0) | (3.0, 6.0) |
|  | MMSE Visual Construction  (0-1) | **Mean**  **(SD, SEM)** | **0.8**  **(0.5, 0.3)** | **0.7**  **(0.5, 0.1)** | **0.7**  **(0.5, 0.1)** | **0.8**  **(0.4, 0.2)** | **0.3**  **(0.5, 0.2)** |
|  |  | Median  (P25, P75) | 1.0  (0.5, 1.0) | 1.0  (0.0, 1.0) | 1.0  (0.0, 1.0) | 1.0  (1.0, 1.0) | 0.0  (0.0, 1.0) |
|  |  | (Min, Max) | (0.0, 1.0) | (0.0, 1.0) | (0.0, 1.0) | (0.0, 1.0) | (0.0, 1.0) |
|  | RBANS Total  (40-160) | **Mean**  **(SD, SEM)** | **72.8**  **(8.3, 4.2)** | **65.5**  **(10.5, 3.2)** | **67.5**  **(12.4, 2.6)** | **72.0**  **(13.4, 6.0)** | **66.0**  **(11.8, 4.5)** |
|  |  | Median  (P25, P75) | 75.0  (67.0, 78.5) | 64.0  (55.0, 77.0) | 68.5  (57.0, 79.0) | 77.0  (72.0, 80.0) | 62.0  (61.0, 68.0) |
|  |  | (Min, Max) | (61.0, 80.0) | (51.0, 80.0) | (49.0, 88.0) | (49.0, 82.0) | (52.0, 90.0) |
|  | RBANS Delayed Memory  (40-154) | **Mean**  **(SD, SEM)** | **56.0**  **(19.3, 9.7)** | **52.7**  **(13.7, 4.1)** | **47.8**  **(5.9, 1.2)** | **69.0**  **(26.2, 11.7)** | **64.3**  **(21.5, 8.1)** |
|  |  | Median  (P25, P75) | 50.0  (44.0, 68.0) | 48.0  (44.0, 64.0) | 48.0  (44.0, 52.0) | 56.0  (56.0, 91.0) | 64.0  (48.0, 92.0) |
|  |  | (Min, Max) | (40.0, 84.0) | (40.0, 84.0) | (40.0, 60.0) | (40.0, 102.0) | (40.0, 94.0) |
|  | RBANS Visuospatial/ Constructional  (40-154) | **Mean**  **(SD, SEM)** | **101.8**  **(28.8, 14.4)** | **90.4**  **(25.6, 7.7)** | **85.4**  **(19.8, 4.2)** | **79.4**  **(21.1, 9.4)** | **80.3**  **(23.9, 9.0)** |
|  |  | Median  (P25, P75) | 113.0  (82.5, 121.0) | 78.0  (64.0, 121.0) | 84.0  (69.0, 105.0) | 78.0  (62.0, 96.0) | 81.0  (60.0, 102.0) |
|  |  | (Min, Max) | (60.0, 121.0) | (60.0, 126.0) | (50.0, 121.0) | (56.0, 105.0) | (50.0, 116.0) |

ICV, intracranial volume; MMSE, Mini-Mental Status Examination; RBANS, Repeatable Battery for the Assessment of Neuropsychological Status; SD, standard deviation; SEM, standard error of the mean.
