# Supplementary material for: Onset of Alzheimer disease in apolipoprotein ɛ4 carriers is earlier in butyrylcholinesterase K variant carriers
Source: BMC Neurol. 2024 Apr 9;24:116. doi: 10.1186/s12883-024-03611-5 (PMC11003149; doi:10.1186/s12883-024-03611-5)
Supplement: Supplementary file 2 — Additional file 2. Ethics committees approving clinical study. [file 12883_2024_3611_MOESM2_ESM.docx]

## Ethics committees approving clinical study

| Principal Investigator | IRB/Ethic Committee | Reference number |
| --- | --- | --- |
| Dr Albert Ludolph | Ethikkommission der Universität Ulm | 412/16 |
| Dr Siegfried Muhlack | Ethik-Kommission der Ruhr-Universität Bochum | 16-5929 |
| Dr Catherine Mummery | London-Central Research Ethics Committee Manchester HRA Centre | 17/LO/0440 |
| Dr Simon Ducharme | MUHC Neurosciences Research Ethics Board | 2017-3206 |
| Dr Juha Rinne | National Committee on Medical Research Ethics | 73/06.00.01/2017 |
| Dr Ralf Bodenschatz | Ethikkommission der Sächsischen Landesärztekammer | EK-AMG-MCB-155/16-1 |
| Dr Peter Paul de Deyn | Central Committee on Research Involving Human Subjects | NL60032.000.16 |
| Dr Anne Borjesson Hansen | Regionala etikprövningsnämnden i Stockholm Karolinska Institutet i Solna | 2017/300-31 |
| Dr Michael Jonsson | Regionala etikprövningsnämnden i Stockholm Karolinska Institutet i Solna | 2017/300-31 |
| Dr Daniel Blackburn | London-Central Research Ethics Committee Manchester HRA Centre | 17/LO/0440 |
| Dr Anja Schneider | Ethikkommission an der Medizinischen Fakultät der Rheinischen Friedrich‑Wilhelms‑Universität Bonn | 035/18-AMG |
| Dr Phillipus Scheltens | Central Committee on Research Involving Human Subjects | NL60032.000.16 |
