# Supplementary figures and images for: Onset of Alzheimer disease in apolipoprotein ɛ4 carriers is earlier in butyrylcholinesterase K variant carriers
Source: BMC Neurol. 2024 Apr 9;24:116. doi: 10.1186/s12883-024-03611-5 (PMC11003149; doi:10.1186/s12883-024-03611-5)

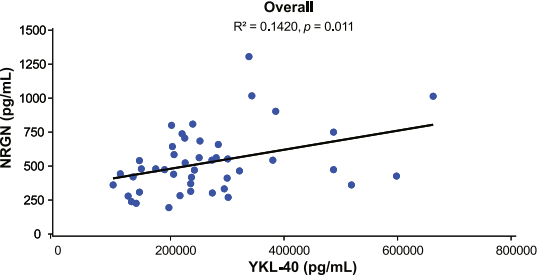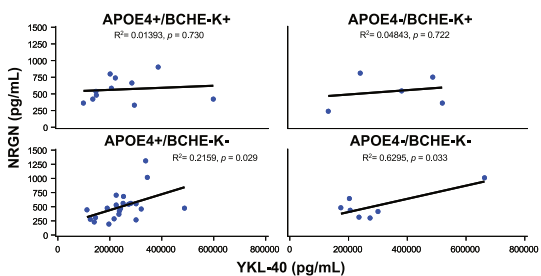

Supplement: Supplementary file 3 — Additional file 3: Figure S1. Correlations in the overall population and in APOE4 and BCHE-K subgroups of Ng versus YKL-40. [file 12883_2024_3611_MOESM3_ESM.pdf]
